# Endoscopic-assisted resection of vestibular schwannomas in high-riding jugular bulb

**DOI:** 10.3171/2021.7.FOCVID2198

**Published:** 2021-10-01

**Authors:** Florian Roser, Tanmoy Maiti, Mohamed Samy Elhammady

**Affiliations:** Department of Neurosurgery, Neurological Institute, Cleveland Clinic Abu Dhabi, United Arab Emirates

**Keywords:** skull base, vestibular schwannoma, endoscopy, high jugular bulb, internal auditory canal, Tubingen line, sitting position, retrosigmoid

## Abstract

The present surgical video demonstrates safe opening of the internal auditory canal (IAC) during vestibular schwannoma surgery via a retrosigmoid approach in the sitting position. Resection of the intrameatal portion of a tumor is important for progression-free survival. Preoperative thin-sliced CT revealed a high-riding jugular bulb obscuring the trajectory. After dural opening, the IAC was approached anteriorly and superiorly. The posterior margin of IAC drilling was above the Tubingen line. Drilling was performed under continuous jugular compression. The vein was pushed down to augment visibility. An angled endoscope was helpful. IAC can be drilled safely in a high-riding jugular bulb with the technique mentioned in the video.

The video can be found here: https://stream.cadmore.media/r10.3171/2021.7.FOCVID2198

## Transcript

**0:21** This video demonstrates endoscopic-assisted resection of vestibular schwannoma (VS) in high-riding jugular bulb (HGB).

**0:28** The MRI demonstrates a T2 VS in dumbbell configuration of right internal auditory canal (IAC) in cerebellopontine angle. The CT scan confirms the remodeling of the bone and high-riding jugular bulb in relation to the IAC, the semicircular ducts, and the cochlea. The HGB is in the trajectory in the way of drilling of IAC from retrosigmoid approach.

**1:03** The patient experienced tumor growth on serial MRI and progressive hearing loss on audiogram over the years.

**1:19** The patient opted for microsurgical resection after discussing the pros and cons of microsurgical resection and radiosurgery. Among the microsurgical approaches, retrosigmoid approach, middle fossa approach, translabyrinthine approach were considered. However, because of dumbbell configuration of IAC, the inferior tumor part would be relatively easier to reach via retrosigmoid approach. The retrosigmoid approach at supine position was not chosen due to the expected venous bleeding during drilling of IAC.

**1:41** The key steps for semisitting position include the pre- and intraoperative transesophageal echo to detect early air entry, as well as central lines for air aspiration, if there is any. Continuous intraoperative neuromonitoring includes SSEP, MEP from positioning, followed by evoked potential as well as facial EMG recordings.

**2:03** For drilling of internal auditory canal, we recommend using only diamond drill to attempt hearing preservation.

**2:14** Drilling of high-riding jugular bulb is done under jugular compression. The drilling is performed with downsizing diamond burrs. The drilling is more limited than usual, and the superior part of the IAC is drilled. For the intracanalicular part, a 30° endoscope is used.

**2:30** The suction irrigation device is demonstrated and tested.

**2:33** The final skin initial incision is marked. After retrosigmoid skin incision, the dissection of muscles and waxing of mastoid air cell to avoid air entry in the sitting position. Preservation of muscle was performed to seal the IAC later on.

**2:55** Osteoplastic craniotomy after dissection of the dura at the sinus angle was performed. Care should be taken at the sigmoid sinus, as the dura is frequently very thin in that area.

**3:13** After dural incision under the microscope, release of CSF was performed. After cerebellar retraction under gravity, cerebellopontine angle comes into view.

**3:25** The anticipated location of the IAC is outlined, as well as the high jugular bulb. This can be seen as the discoloration of the bone just posterior to the IAC. The Tubingen line marks the inferior border of the opening of the IAC, to avoid the drilling of the endolymphatic sac. After removal of the dura over the temporal bone, the discoloration and location of high jugular bulb is visible more clearly, as there was very thin bone overlying the sinus.

**4:20** The drilling of the IAC was performed with diamond burrs of decreasing sizes under controlled and simultaneous bilateral jugular compression by the anesthesiologist to avoid the air entry in case of drilling and injury into the jugular bulb. The direction of drilling is superior-anterior toward inferior-posterior to open the anterior part of the IAC first, and then around the high jugular bulb at the posterior edge.

**5:22** After sufficient opening of the IAC, the vestibular schwannoma was partially resected with microsurgical and bimanual techniques until the remaining intracanalicular part.

**5:40** The dumbbell-shaped widened IAC was approached by a 30° endoscope.

**5:50** Here we see the anatomical location of the seventh nerve separated by the transverse crest from the superior vestibular nerve and the high-riding jugular bulb at the inferior posterior area of the temporal bone.

**6:10** With standard microsurgical instruments, the remaining tumor was mobilized first from the nerve and then from the enlarged cavity to achieve complete resection.

**6:29** This is performed under continuous electrophysiological monitoring including brainstem auditory evoked potential, as well as facial nerve EMG and MEPs.

**6:48** In this safe zone away from the nerve, the remaining tumor part in this excavated IAC can be mobilized and subsequently removed.

**7:08** Following tumor resection, the internal auditory canal was filled with the muscle patch and fibrin glue.

**7:16** The postoperative CT scan shows opening of internal auditory canal with preserved semicircular ducts.

**7:23** The postoperative audiogram shows preserved hearing with mild deterioration in higher frequencies. The patient was discharged with full facial nerve integrity and hearing preservation on postoperative day 5. Thank you very much for your attention.

**7:30 References** ^1–7^

## Acknowledgments

We acknowledge Ms. Sony Thankachan for her help with preparing the illustrations.

## Disclosures

The authors report no conflict of interest concerning the materials or methods used in this study or the findings specified in this publication.

## Author Contributions

Primary surgeon: Roser. Editing and drafting the video and abstract: all authors. Critically revising the work: all authors. Reviewed submitted version of the work: all authors. Approved the final version of the work on behalf of all authors: Roser. Supervision: Elhammady.
